# Transhepatic Access for Left Atrial Appendage Closure in a Patient With Inferior Vena Cava Interruption

**DOI:** 10.1016/j.jaccas.2025.106614

**Published:** 2026-01-21

**Authors:** Pablo D. Liva, Guillermo Eisele, Marcelo A. Agüero, Esteban Villegas, Gastón Pozzi, Pedro Zambrano-Rodas, Gabriela Ríos, Adolfo G. López-Campanher, Matías J. Arévalo, Jorge A. Baccaro

**Affiliations:** a Department of Interventional Cardiology, Instituto de Cardiología de Corrientes “Juana Francisca Cabral”, Corrientes, Argentina; b Department of Interventional Radiology, Children's Hospital “Ricardo Gutierrez”, Buenos Aires, Argentina

**Keywords:** atrial fibrillation, inferior vena cava interruption, left atrial appendage occlusion, percutaneous closure, structural heart intervention, transhepatic access

## Abstract

**Background:**

Left atrial appendage occlusion (LAAO) is an alternative stroke-prevention strategy in patients with nonvalvular atrial fibrillation who have contraindications to oral anticoagulation. Femoral venous access is the standard approach.

**Case Summary:**

We report a 78-year-old woman with nonvalvular atrial fibrillation, high bleeding risk due to recurrent falls, and prior traumatic subdural hematoma, referred for LAAO. The initial procedure attempt was aborted after intraprocedural discovery of congenital inferior vena cava interruption, precluding femoral access. In a second stage, the procedure was successfully completed via transhepatic venous access without complications.

**Discussion:**

This case highlights the feasibility of transhepatic access for structural heart interventions in patients with complex venous anatomy.

**Take-Home Messages:**

Transhepatic access is a safe alternative for LAAO in patients with inferior vena cava interruption. Preprocedural imaging and flexible access strategies are essential in structural interventions for patients with complex venous anatomy.

## Visual Summary

**Figure:**
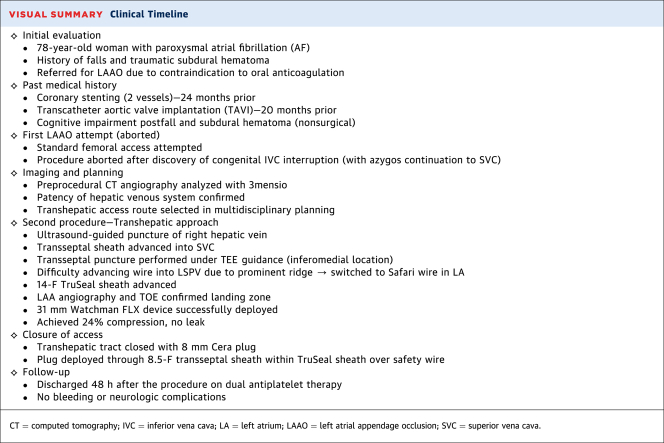

Percutaneous left atrial appendage occlusion (LAAO) has become an established and effective strategy for stroke prevention in patients with nonvalvular atrial fibrillation who have contraindications to oral anticoagulation or are at high bleeding risk. Femoral venous access remains the preferred route for the procedure, providing direct and appropriately angled access to the left atrium via transseptal puncture.^1^^,^^2^

**Visual Summary:**
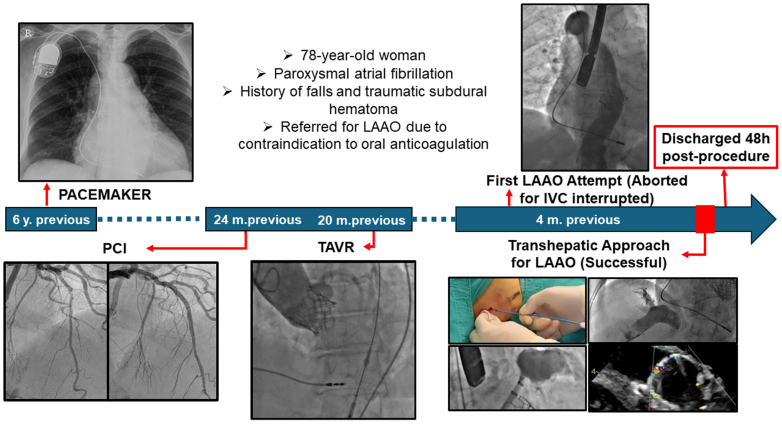
Timeline IVC = inferior vena cava; LAAO = left atrial appendage occlusion; PCI = percutaneous coronary intervention; TAVR = transcatheter aortic valve replacement.

However, in rare cases of congenital vascular anomalies such as complete interruption of the inferior vena cava (IVC), femoral access is not feasible. In these patients, transhepatic venous access emerges as a viable and safe alternative, enabling successful structural heart interventions. Although less commonly used, this technique has been mainly described in pediatric cardiology and complex adult interventions with atypical vascular anatomy.^3^, ^4^, ^5^

We report a case of successful percutaneous LAAO via transhepatic venous access in a patient with congenital complete interruption of the IVC highlighting the importance of preprocedural planning and flexibility in vascular access selection.

## History of Presentation

A 78-year-old female patient with paroxysmal atrial fibrillation and contraindication oral anticoagulation was referred for LAAO (Table 1). Physical examination was unremarkable.

**TABLE 1:** Clinical Timeline

• Initial evaluation ○ 78-year-old woman with paroxysmal atrial fibrillation (AF) ○ History of falls and traumatic subdural hematoma ○ Referred for LAAO due to contraindication to oral anticoagulation • Past medical history ○ Coronary stenting (2 vessels)—24 months prior ○ Transcatheter aortic valve implantation (TAVI)—20 months prior ○ Cognitive impairment postfall and subdural hematoma (nonsurgical) • First LAAO attempt (aborted) ○ Standard femoral access attempted ○ Procedure aborted after discovery of congenital IVC interruption (with azygos continuation to SVC) • Imaging and planning ○ Preprocedural CT angiography analyzed with 3mensio ○ Patency of hepatic venous system confirmed ○ Transhepatic access route selected in multidisciplinary planning • Second procedure—Transhepatic approach ○ Ultrasound-guided puncture of right hepatic vein ○ Transseptal sheath advanced into SVC ○ Transseptal puncture performed under TEE guidance (inferomedial location) ○ Difficulty advancing wire into LSPV due to prominent ridge → switched to Safari wire in LA ○ 14-F TruSeal sheath advanced ○ LAA angiography and TOE confirmed landing zone ○ 31 mm Watchman FLX device successfully deployed ○ Achieved 24% compression, no leak • Closure of access ○ Transhepatic tract closed with 8 mm Cera plug ○ Plug deployed through 8.5-F transseptal sheath within TruSeal sheath over safety wire • Follow-up ○ Discharged 48 h after the procedure on dual antiplatelet therapy ○ No bleeding or neurologic complications

CT = computed tomography; IVC = inferior vena cava; LA = left atrium; LAAO = left atrial appendage occlusion; LSPV = left superior pulmonary vein; SVC = superior vena cava; TEE = transesophageal echocardiogram.

## Past Medical History

The patient was relatively frail, with a history of two-vessel coronary angioplasty with stent placement 24 months earlier, and transcatheter balloon-expandable aortic valve implantation 20 months ago.

She also had a history of hospitalization at another institution due to cognitive disturbances following a fall. A subdural hematoma was diagnosed and managed conservatively. The hematoma evolved without the need for surgical intervention.

**Figure 1:**
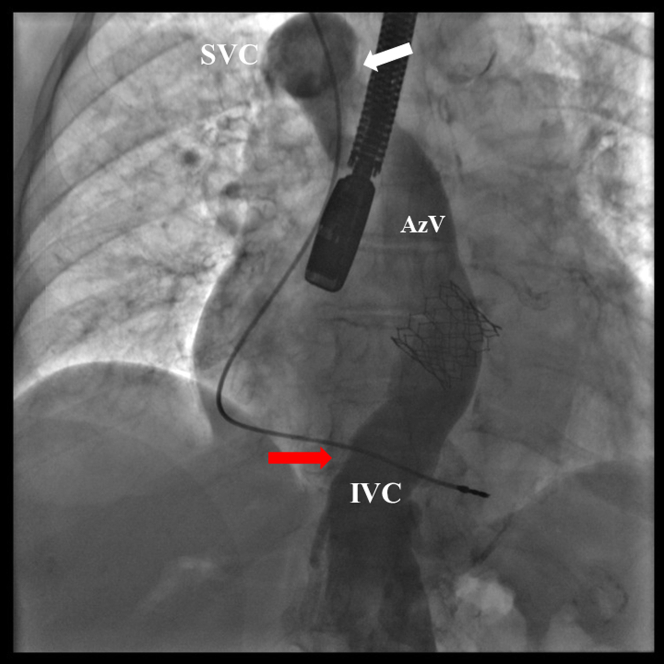
Cavography Venous angiography showing an IVC interruption above renal vein (red arrow). AzV draining into SVC (white arrow). AzV = azygos vein; IVC = inferior vena cava; SVC = superior vena cava.

An initial attempt at LAAO was aborted due to the unexpected identification of a congenital interruption of the IVC above the renal veins, with venous drainage to the superior vena cava via the azygos vein (Figure 1, Video 1).

## Management

Contrast-enhanced computed tomography demonstrated patency of the suprahepatic vein.

After multidisciplinary discussion and consultation with an interventional radiologist, a second attempt at left atrial appendage closure was planned 50 days after the initial attempt.

**Figure 2:**
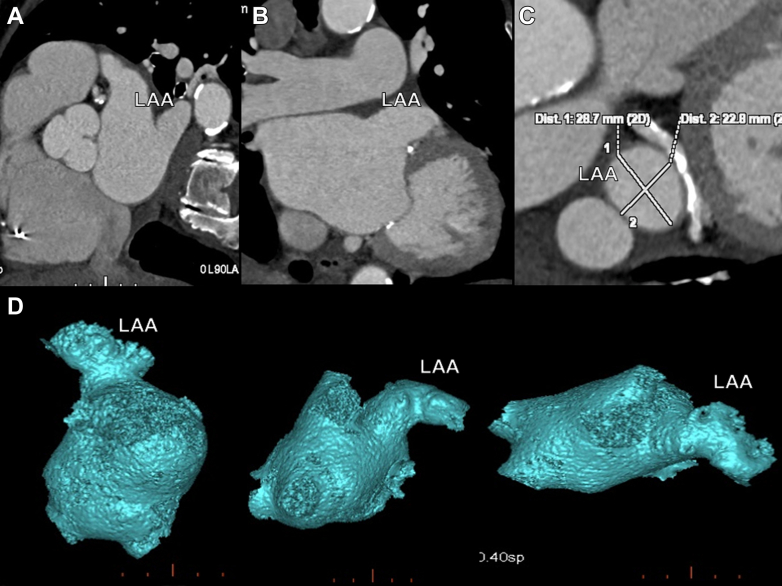
Preprocedural Computed Tomography Angiography (A to C) Multiplanar CTA views showing LAA morphology and excluding thrombus. (D) Three-dimensional reconstruction of LAA. CTA = computed tomography angiography; LAA = left atrial appendage.

The computed tomography scan was used to assess the morphology of the left atrial appendage and to guide both device sizing and the optimal location for transseptal puncture (Figure 2).

**Figure 3:**
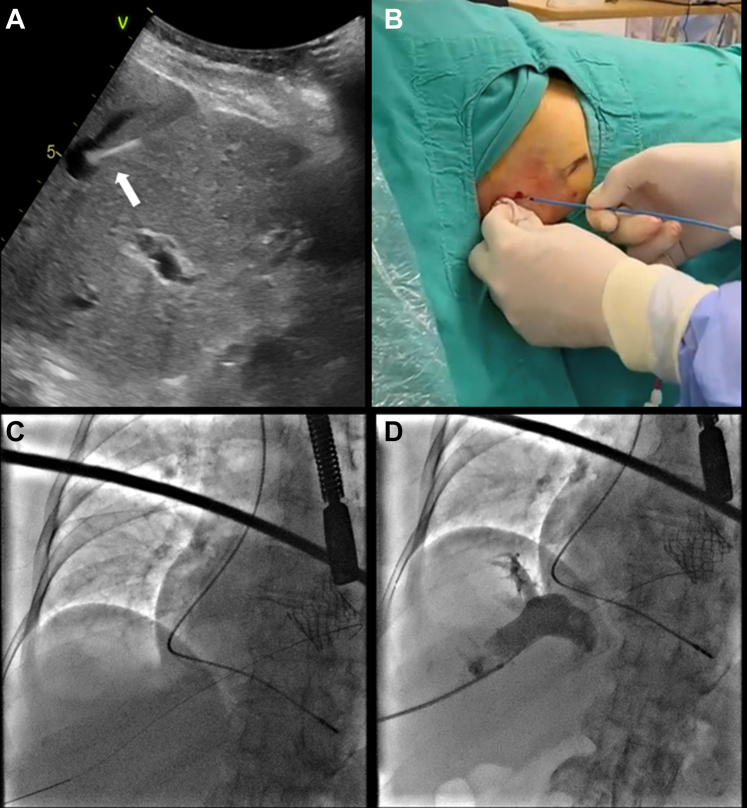
Suprahepatic Venous Access (A) Ultrasound-guided percutaneous puncture of SHV (white arrow) and (B) dilation with 4-F catheter. (C) Guidewire progression to SHV-RA junction. (D) Venogram confirming adequate access. RA = right atrium; SHV = suprahepatic vein.

Under general anesthesia with orotracheal intubation, skin incision was made above the 12th rib at the midaxillary line. Under echo and fluoroscopic guidance, a micropuncture needle achieved suprahepatic vein, and a 0.018-inch wire (Neff Set D'Agostino Modification Cook Medical) was advanced, aiming toward the IVC–right atrial junction (Videos 2 and 3). Hepatic venous access was confirmed with aspiration of venous blood and venogram with a contrast injection (Figure 3, Video 4). Half dose of heparin was administered before transseptal puncture. A J tipped wire with a Right Judkin catheter were advanced into the superior vena cava. A transseptal sheath (Fixed Curve Transseptal Sheath 60 cm, 30֯ [Merit Medical System Utah]) was advanced over the wire in the superior vena cava.

**Figure 4:**
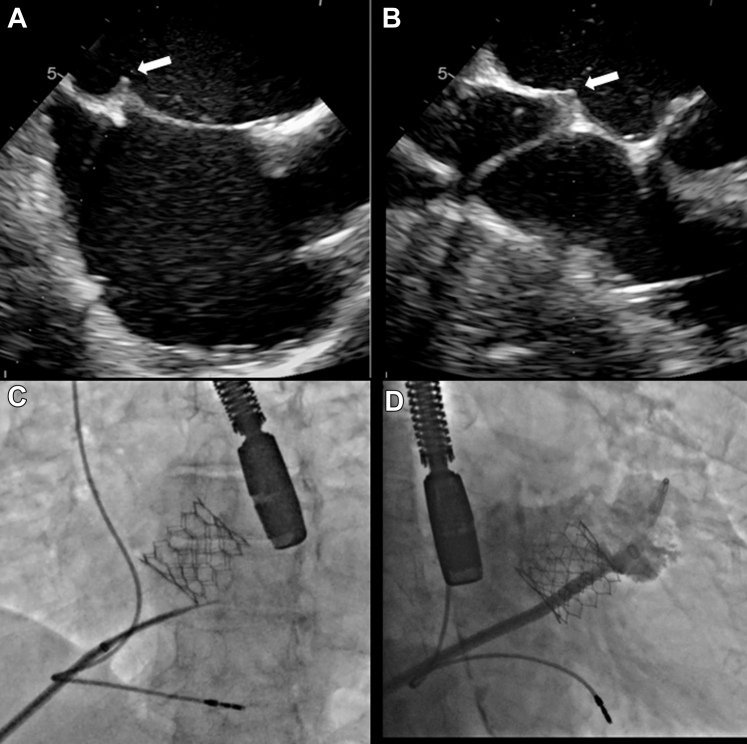
TEE-Guided Transseptal Puncture (A) TEE bicaval and (B) SAX views showing sheath tenting (white arrows). (C) X-ray fluoroscopy image with the septal puncture needle positioned. (D) LAA angiography. LAA = left atrial appendage; SAX = short axis; TEE = transesophageal echocardiography.

Transseptal puncture was done under transesophageal echocardiography (TEE) guidance. The original M curve of the transseptal needle (EverPace, Microport Medical, Medtech Co Shanghai) was manually slightly straightened to facilitate coaxial alignment with the interatrial septum from the transhepatic access. Maneuverability of the sheath to obtain an inferoposterior puncture location was achieved without difficulty (Figure 4, Video 5). Once the left atrium was achieved, a second bolus of intravenous heparin was given to complete 100 units per kilogram to achieve an activated clotting time above 250 seconds.

**Figure 5:**
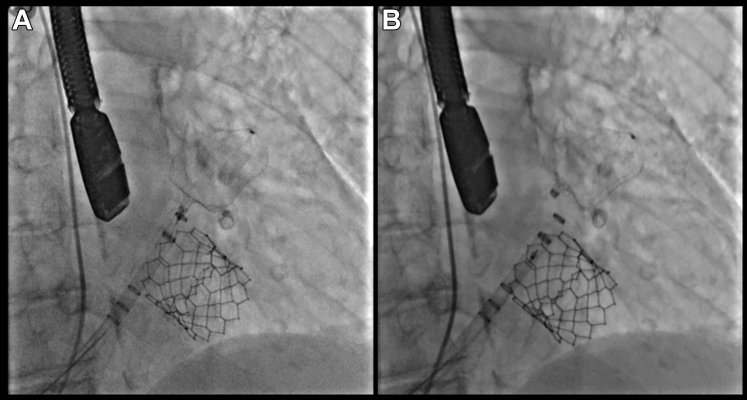
Watchman FLX Deployed (A) Watchman placement in accurate position before to deployment. (B) Post delivered.

Advancement of the guidewire into the left superior pulmonary vein was unsuccessful due to the presence of a prominent pulmonary vein ridge. As a result, a decision was made to perform a wire exchange over a Safari small curve wire positioned in the left atrium. Predilation of the hepatic tract with a 14-F dilator was required to facilitate advancement of the 14-F Watchman Access Double curve (Boston Scientific Truseal Access System) (Video 6). Once the sheath was successfully advanced, the subsequent steps proceeded as usual. Left atrial appendage angiography was performed in right anterior oblique caudal and cranial projections (25°/25°) (Figure 4D). Measurements were confirmed by both TEE and angiography. A 31-mm Watchman FLX device (Boston Scientific) was successfully deployed, achieving 26% compression with no residual leak observed (Figure 5, Video 7).

**Figure 6:**
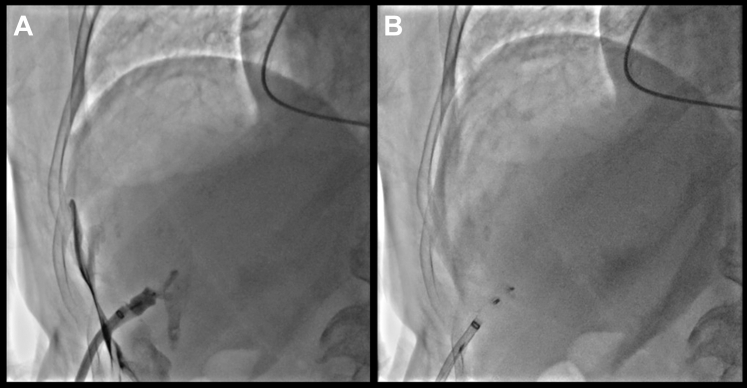
Transhepatic Access Closure (A) Fluoroscopic view to verify accurate deployment of Cera vascular plug system at capsular plain. (B) Final plug site.

Hemostasis of the transhepatic venous access site was achieved using an 8-mm Cera Vascular Plug System (Lifetech Scientific). To deploy the plug, the 8.5-F transseptal sheath was advanced through the 14-F TruSeal sheath over a safety guidewire positioned in the hepatic vein. With gradual sheath withdrawal and contrast injections, the vessel wall was clearly identified, allowing accurate deployment of the closure device at the appropriate site (Figure 6, Videos 8 and 9).

## Outcomes and Follow-Up

The procedure was completed successfully without intraprocedural or postprocedural complications. Final TEE confirmed appropriate device position, adequate compression, and complete sealing of the left atrial appendage, with no peridevice leak or device-related thrombus (Video 7). The patient remained hemodynamically stable and was discharged 48 hours later on dual antiplatelet therapy. The patient reported no bleeding or neurologic events.

## Discussion

Percutaneous LAAO has emerged as a valuable stroke-prevention strategy for patients with nonvalvular atrial fibrillation who have contraindications to long-term oral anticoagulation. The standard femoral venous approach is effective in the vast majority of cases, providing a direct and familiar pathway for transseptal access. However, anatomical variants such as congenital interruption of the IVC add considerable challenge to this conventional access. In such cases, an alternative venous approach must be considered. Transhepatic venous access, though technically more demanding, provides a feasible and safe alternative for structural cardiac procedures when femoral and jugular access are not viable. Although traditionally used in pediatric and interventional radiology procedures, this unusual approach is emerging as a valuable alternative in adult structural heart interventions when conventional access is unavailable.^6^

Tandon et al^7^ and Quiroz Alfaro et al^8^ demonstrated the successful use of transhepatic access for LAAO in a patient with interrupted IVC, reinforcing the utility of this approach in rare anatomical settings. Our case adds to this growing body of evidence by showing that careful preprocedural planning can facilitate the safe execution of transhepatic LAAO even in relatively frail elderly patients.

Key technical considerations include real-time ultrasound guidance for hepatic vein puncture, selection of an appropriate transseptal sheath, and in our case, a slightly straightened transseptal needle to achieve the perpendicular septal angle and secure exchange over a stable wire to allow device delivery. As in previous reports, we found that with proper planning and a multidisciplinary approach, outcomes with this access route can mirror those of standard techniques.

Importantly, the transhepatic approach should not be reserved only as a salvage option but considered proactively in patients with known IVC interruption. The ability to adapt sheath selection and transseptal puncture strategy based on multiplanar reconstruction imaging as we did using 3mensio software was critical to procedural success.

## Conclusions

This case highlights the feasibility and safety of transhepatic venous access for LAAO in patients with congenital interruption of the IVC. With careful preprocedural planning, appropriate imaging, and close collaboration between interventional cardiology and radiology teams, this alternative access route can be successfully employed to achieve outcomes comparable to conventional femoral access. Transhepatic access should be considered a valid and reproducible option in anatomically complex patients who are otherwise eligible for structural heart interventions.

## Funding Support and Author Disclosures

Dr Liva serves as a proctor and speaker for Boston Scientific regarding Watchman devices. Dr Eisele has received honoraria for participating in hepatic access procedures. All other authors have reported that they have no relationships relevant to the contents of this paper to disclose.

**Take-Home Messages**

• Transhepatic venous access is a viable and safe alternative for left atrial appendage occlusion in patients with congenital inferior vena cava interruption.
• Comprehensive preprocedural imaging and a collaborative team approach are essential for optimizing procedural planning and ensuring successful outcomes.

## Appendix

Video 1 Cavography Showing Inferior Vena Cava Interruption ICVinterruption. Azigos vein draining into superior vena cava.

Video 2 Echo Guidance Venous Suprahepatic Puncture

Video 3 Micro Wire Advanced Into Hepatic Vein

Video 4 4-F Sheath and Venogram to Confirm Hepatic Venous Access

Video 5 Transseptal Sheath Advanced Into Left Atrium

Video 6 Dilation of the Intrahepatic Tract Was Performed With a 14-F Dilator Advanced Over a Safari Wire to Facilitate Sheath Insertion

Video 7 Watchman Flex 31 mm Deployed With No Leak or Thrombus

Video 8 At the End of the Procedure, an 8-mm Cera Plug Was Deployed in the Hepatic Tract to Achieve Hemostasis

Video 9 Last Injection Showing Capsular Plane and Plug
